# Healthcare professionals’ views on patient-centered care in hospitals

**DOI:** 10.1186/s12913-015-1049-z

**Published:** 2015-09-16

**Authors:** Mathilde Berghout, Job van Exel, Laszlo Leensvaart, Jane M. Cramm

**Affiliations:** Institute of Health Policy and Management, Erasmus University Rotterdam, Rotterdam, The Netherlands

**Keywords:** Patient-centered care, Quality of care, Healthcare professionals, Q methodology, Hospital

## Abstract

**Background:**

Patient-centered care (PCC) is a main determinant of care quality. Research has shown that PCC is a multi-dimensional concept, and organizations that provide PCC well report better patient and organizational outcomes. However, little is known about the relative importance of PCC dimensions. The aim of this study was therefore to investigate the relative importance of the eight dimensions of PCC according to hospital-based healthcare professionals, and examine whether their viewpoints are determined by context.

**Methods:**

Thirty-four healthcare professionals (16 from the geriatrics department, 15 from a surgical intensive care unit, 3 quality employees) working at a large teaching hospital in New York City were interviewed using Q methodology. Participants were asked to rank 35 statements representing eight dimensions of PCC extracted from the literature: patient preferences, physical comfort, coordination of care, emotional support, access to care, continuity and transition, information and education and family and friends. By-person factor analysis was used to reveal patterns of communality in statement rankings, which were interpreted and described as distinct viewpoints.

**Results:**

Three main viewpoints on elements important for PCC were identified: “treating patients with dignity and respect,” “an interdisciplinary approach” and “equal access and good outcomes.” In these viewpoints, not all dimensions were equally important for PCC. Furthermore, the relative importance of the dimensions differed between departments. Context thus appeared to affect the relative importance of PCC dimensions.

**Conclusion:**

Healthcare organizations wishing to improve PCC should consider the relative importance of PCC dimensions in their specific context of care provision, which may help to improve levels of patient-centeredness in a more efficient and focused manner. However, as the study sample is not representative and consisted only of professionals (not patients), the results cannot be generalized outside the sample. More research is needed to confirm our study findings.

## Background

Since the Institute of Medicine described patient-centered care (PCC) as one of the six most important determinants of quality of care – along with safe, effective, timely, efficient and equitable care – PCC has received much more attention [1]. Richardson and colleagues [1] defined PCC as care that is “respectful of and responsive to individual patient preferences, needs, and values, and ensuring that patient values guide all clinical decisions.” PCC has been shown to result in improved health outcomes, including survival, greater patient satisfaction and well-being [2]. Furthermore, PCC is related to improved communication between patients and healthcare professionals, and it has been associated with reductions in healthcare resource needs and costs [1, 3–6]. The Picker Institute identified eight dimensions of PCC: (a) respect for patient preferences, values and expressed needs; (b) information, education and communication; (c) coordination and integration of care and services; (d) emotional support; (e) physical comfort; (f) involvement of family and friends; (g) continuity and transition; and (h) access to care and services [7, 8]. Although constellations of these eight PCC dimensions are known to lead to better outcomes [2], whether every dimension contributes equally remains unclear. Furthermore, healthcare professionals’ perceptions about PCC and whether these perceptions are determined by context (which appears to affect the relationship between PCC and outcomes) are not well known [2, 9, 10]. However, an understanding of these perceptions is essential to improve the quality of care, as they are known to predict care quality [11] and healthcare professionals play an integral role in delivering PCC [12, 13]. More knowledge about the relative importance of PCC dimensions can also contribute to decision making about investment in PCC while delivering healthcare in an economic context of limited financial resources. Thus, the aim of this study was to investigate the relative importance of the eight dimensions of PCC from the perspectives of healthcare professionals, and to examine whether their views are determined by context.

## Methods

Healthcare professionals’ views about PCC were explored using Q methodology, which combines qualitative and quantitative techniques for the systematic study of subjectivity [14–16]. In a Q-methodological study, participants are generally presented with a set of statements about the study topic and instructed to rank these statements according to, for example, agreement, importance or preference, and to explain their ranking. The underlying assumption is that by ordering the statements, participants reveal their subjective viewpoints about the study subject, and that correlation between rankings indicates similarity of viewpoint. By-person factor analysis [17] is then used to identify subgroups of like-minded participants. The resulting factors are interpreted and described as shared views on the subject of study. These quantitative data are supplemented with qualitative data obtained from participants’ explanations of their rankings during interviews.

Q methodology differs from other methods involving factor or cluster analysis in its focus on studying subjectivity (i.e., by examining correlations among people), rather than objectivity (i.e., by examining correlations among items). The statements are sampled for to achieve representativeness of the study topic, and the participants are sampled purposively to ensure diversity (much like instruments in a common survey). The results of a Q-methodological study can thus be generalized to the subject of study, which is the population from which the statements were sampled, but not to the population of participants. In this study, participants were asked to rank statements representing PCC dimensions according to their importance for PCC.

The institutional review board for human subject research of The Mount Sinai Hospital, New York, approved the study protocol (no. 14–00342).

### Development of the statement set

The authors developed the statement set for use in different care settings (e.g., hospitals, outpatient clinics) and contexts (e.g., departments, care pathways), and with different stakeholder groups (e.g., patients, professionals). As described in Cramm et al. [18], the eight previously defined dimensions of PCC [7, 8] served as a starting point for the development of the research instrument, and additional literature on PCC [9, 10, 19–21] revealed no additional PCC dimension that should be considered. In an iterative process involving all authors, a set of 35 opinion statements was developed (Table 1). To test the comprehensibility of the statements and their applicability to the situation, a pilot study using the same strategy as the overall study was conducted with five healthcare professionals (an internist, a surgical oncologist, two anesthesiologists, and an oncology nurse) working in the study setting. Given the positive results of the pilot study (all items were clear and no aspect was missed), no change to the statement set was made.

**Table 1:** Statement set

Dimension of PCC	Examples	Statements
Patients’ preferences	- Providing care in a respectful atmosphere with dignity and respect	1. Healthcare professionals treat patients with dignity and respect.
	- Focus on quality of life issues / whole-person care	2. Healthcare is focused on improving patients’ quality of life.
		3. Healthcare professionals take patients’ preferences into account.
	- Informed and shared decision making / patient participation and involvement	4. Healthcare professionals involve patients in decisions about their care.
	- Personal goals and outcomes	5. Patients are supported in setting and achieving their own treatment goals.
Physical comfort	- Pain management	6. Healthcare professionals pay attention to pain management.
	- Assistance with daily living needs	7. Healthcare professionals take patients’ preferences for support and daily living needs into account.
	- Hospital surroundings and environment	8. Patient areas in hospital are clean and comfortable.
		9. Patients have privacy in the hospital.
Coordination of care	- Coordination and integration of care	10. Healthcare professionals are well informed; patients need to tell their story only once.
		11. Patient care is well coordinated among professionals.
	- Spokesperson for navigation through the system	12. Patients know who is coordinating their care.
		13. Patients have a primary contact who knows everything about their condition and treatment.
	- Teamwork	14. Healthcare professionals work as a team in care delivery to patients.
Emotional support	- Anxiety about consequences of the changed situation	15. Healthcare professionals pay attention to patients’ anxiety about their situations.
	- Creating support systems	16. Healthcare professionals involve relatives in emotional support of the patient.
	- Anxiety about the impact of one’s illness on one’s family and loved ones	17. Healthcare professionals pay attention to patients’ anxiety about the impact of their illness on their loved ones.
Access to care	- Access to location / specialist	18. The hospital is accessible for all patients.
	- Availability of transportation	19. Clear directions are provided to and inside the hospital.
	- Clear instructions provided on how and when to get referral	
	- Ease of scheduling appointments	20. Appointment scheduling is easy.
	- Waiting time	21. Waiting times for appointments are acceptable.
	- Language barrier	22. Language is not a barrier to access to care.
	- Cultural differences	
Continuity and transition	- Understandable, detailed information regarding all aspects of care	23. When a patient is transferred to another ward, relevant patient information is also transferred.
	- Coordination and planning of ongoing treatment	24. Patients who are transferred are well informed about where they are going, what care they will receive, and who their contact person will be.
	- Provide information regarding access to support after hospital discharge	25. Patients receive skilled advice about care and support at home after hospital discharge.
Information and education	- Information on all aspects of care (e.g., clinical status, progress, prognosis, care processes)	26. Patients are well informed about all aspects of their care.
	- Information on processes of care	27. Patients can access their care records.
	- Information and education to facilitate autonomy and self-care	28. Patients are in charge of their own care.
		29. Healthcare professionals support patients to be in charge of their care.
	- Open communication between patient and caregiver	30. Open communication between patients and healthcare professionals occurs.
	- Skills and knowledge of caregiver	31. Healthcare professionals have good communication skills.
Family and friends	- Accommodations	32. Accommodations for relatives are provided in or near the hospital.
	- Respect for role in decision making	33. Healthcare professionals involve relatives in decisions about the patient’s care.
	- Support for family as caregivers	34. Healthcare professionals pay attention to loved ones in their role as the patient’s caregivers.
	- Recognition of the needs of family and friends	35. Healthcare professionals pay attention to the needs of the patient’s family and friends.

*PCC* Patient-centered care

Source: [18]

### Interviews

Semi-structured interviews were conducted with 34 healthcare professionals working at the Mount Sinai Hospital, a large teaching hospital in New York City committed to PCC delivery [22]. Healthcare professionals from the geriatrics department and the surgical intensive care unit (SICU) were invited to participate in this study. These departments where chosen because many attending patients have complex health issues, a situation in which PCC is expected to be beneficial. Moreover, these departments represent two very distinct caregiving contexts, potentially leading to different views on PCC.

A representative sample of healthcare professionals was interviewed; this sample comprised physicians (*n* = 11), nurses (*n* = 8), quality employees (*n* = 3), managers (*n* = 2) and others (*n* = 10; Table 2). These professionals were approached at their workplaces and asked whether they were willing to participate. Those who had worked directly with patients for at least 3 years, allowing for the development of meaningful viewpoints about PCC, were included. Study participation was concluded when new information was no longer revealed during consecutive interviews.

**Table 2:** Sample characteristics

Characteristic	Surgical ICU (*n* = 15)	Geriatrics (*n* = 16)	Quality employees (*n* = 3)	Total (*n* = 34)
Sex (% female)	53	88	100	74
Mean age (years)	41	41	52	45
Profession				
Physician	5	6	0	11
Nurse	5	3	0	8
Manager	1	1	0	2
Quality employee	0	0	3	3
Other^a^	4	6	0	10
Mean duration of employment (years)	15	12	14	14
Mean time working directly with patients (years)	17	14	12	14

*ICU* Intensive care unit

^a^Nurse practitioners (2), physician’s assistant (1), nutritionists (3), social workers (2), medical assistant (1), patient care technician (1)

**Fig. 1:**
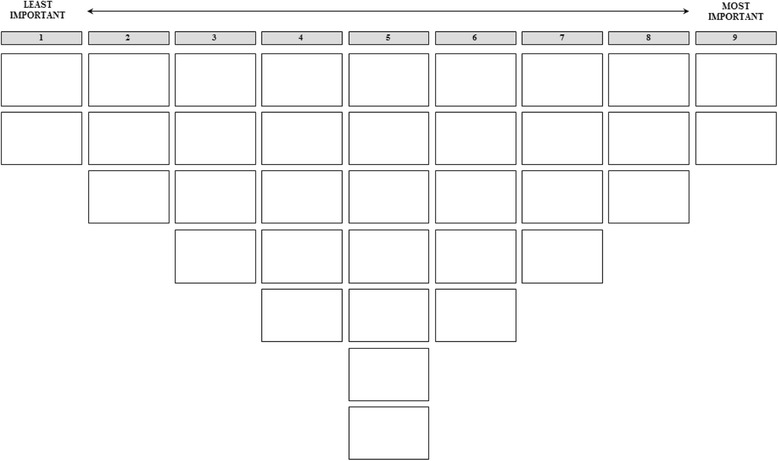
Score sheet

During the interviews, respondents were presented with the set of 35 statements, printed on cards, in random order. Respondents were first asked to read all statements carefully and sort them into three piles representing aspects that they considered important, neutral/not relevant and unimportant for PCC. Respondents were then asked to read the statements in each pile again and to rank them from least to most important for PCC using a score sheet (Fig. 1). The score sheet is a prearranged frequency distribution that forces all respondents to rank the statements using a single format, which is as standard way to delineate and standardize the data collection procedure [16]. The range and steepness of the distribution were chosen following common guidelines: the range from −4 to +4 is considerable comfortable for respondents considering the number of statements (*n* = 35); a steeper distribution is considered appropriate for topics of higher complexity [16]. After ranking the statements, respondents were asked to elaborate on their choices and to explain the motivations underlying their ranking of the two most important and two least important statements. Their answers were recorded and transcribed, and were used to aid interpretation and description of the results.

### Analysis

The ranking data were entered in by-person factor analysis using common techniques in Q methodology (i.e., centroid factor extraction, followed by varimax rotation). Based on the criteria that a factor should have an Eigenvalue larger than 1 and be defined by at least two respondents, the data supported a maximum of four factors, explaining 57 % of the variance in the data. The three-factor solution, explaining 54 % of the variance, was selected as most comprehensible and best interpretable. These factors were defined by 14, 9 and 7 respondents and explained 23, 16 and 15 % of the variance, respectively. For each factor, a composite ranking of the 35 statements was computed based on rankings of respondents that were associated significantly (*p* <.05) with that factor, and distinguishing and consensus statements were identified. Statements with significantly different rankings for a given factor relative to all other factors were considered to be distinguishing for that factor, whereas statements whose rankings did not differ significantly between any pair of factors were defined as consensus statements. Viewpoints of professionals from each department were examined separately in subgroup analyses. Data were analyzed using PQMethod 2.11 software [23].

The resulting factors were interpreted and described as viewpoints on PCC. For each viewpoint, an initial interpretation was drafted based on the quantitative data from composite statement ranking. The interpretations were refined using the distinguishing and consensus statements, as well as qualitative data from respondents’ explanations associated with each factor. To investigate whether PCC viewpoints were determined by context, separate factor analyses were also performed using datasets from the two departments.

## Results

Factor analysis revealed three main viewpoints on PCC. The idealized rankings of statements for these perspectives are presented in Table 3, alongside the results at the department level.

**Table 3:** Idealized ranking of the 35 statements for the full sample and by department

Statements		View 1	View 2	View 3	Geriatrics		Surgical IC Unit		
					View 1	View 2	View 1	View 2	View 3
Patients’ preferences									
1	Healthcare professionals treat patients with dignity and respect	+4	+3	+4	+4	+4	+4	+4	+4
2	Healthcare is focused on improving patients’ quality of life	+2**	+4	+3	+1**	+3	+4*	+3*	0**
3	Healthcare professionals take into account patient preferences	+2*	+1	+1	+3**	+1	+3*	0*	−2**
4	Healthcare professionals involve patients in decisions regarding their care	+4	0**	+3	+2	+3	+3	+2	+3
5	Patients are supported to set and achieve their own treatment goals	+3**	−3**	0*	+2**	−1	+2**	−2	−2
Physical comfort									
6	Healthcare professionals pay attention to pain management	+1	+2	+1	+1	+1	+2	+2	+2
7	Healthcare professionals take patient preferences for support with their daily living needs in to account	−1	0	−1*	0**	−2	−2	−1	−1
8	Patients areas in hospital are clean and comfortable	−2**	0	+1	−3**	+1	−1	0	−1
9	Patients in hospital have privacy	−1	−1	0**	−2**	0	0	−3**	0
Coordination of care									
10	Healthcare professionals are well-informed; patients need to tell their story only once	−2	−3	−2	−3**	−1	−2	−3	−4
11	Patient care is well-coordinated between professionals	+1	+2	+2	+1	+2	0	+1	+2
12	Patients know who is coordinating their care	+3*	+1	+1	+2**	0	+3	- 1**	+2
13	Patients have a first point of contact who knows everything about their condition and treatment	+1**	−2*	0*	+4**	0	0	−1	−3*
14	Healthcare professionals work as a team in care delivery to patients	+1	+4**	+1	+1	+1	+1**	+4	+3
Emotional support									
15	Healthcare professionals pay attention to patients’ anxiety about their situation	0	+1	0	0	0	+1	0	+1
16	Healthcare professionals involve relatives in the emotional support of the patient	−1**	+1**	−2**	−1	−2	−1	+2**	−1
17	Healthcare professionals pay attention to patients’ anxiety about the impact of their illness on their loved ones	0	0	−2**	0*	−1	−1	−2	+1*
Access to care									
18	The hospital is accessible for all patients	−2**	−1**	+4**	−3**	+4	0	+2**	0
19	Clear directions are provided to and inside the hospital	−4	−2*	−3	−4	−3	−3	0*	−3
20	It is easy to schedule an appointment	−3**	−2	−1	−1	0	−3*	−2	−2
21	Waiting times for an appointment are acceptable	−3	−2	−3	−2	−2	−4**	−2	0
22	Language is not a barrier for access to care	0	0	+2**	0**	+2	+1	+1	+1
Continuity and transition									
23	When a patient is transferred to another ward, relevant patient information is transferred as well	0	+2**	0	0*	+1	0**	+3	+2
24	Patients who are transferred are well-informed about where they are going, what care they will receive and who will be their contact person	0	0	0	0	0	0	0	0
25	Patients get skilled advice about care and support at home after hospital discharge	−1	0	−1	−1	−1	−1	0	0
Information and education									
26	Patients are well-informed about all aspects of their care	+3	+2	+3*	+2	+3	+2	+3	+3
27	Patients can access their care records	−3	−1**	−3	−2	−3	−4**	+1**	−2**
28	Patients are in charge of their own care	0*	−4**	−1*	−1**	−2	−2**	−4	−4
29	Healthcare professionals support patients to be in charge of their care	+1**	−3**	0**	+1**	−3	+1	−3**	0
30	There is open communication between patient and healthcare professionals	+2	+3	+2	+3	+2	+1	+1	+4**
31	Healthcare professionals have good communication skills	+2	+3	+2	+3	+2	+2	0	+1
Family and friends									
32	Accommodation for relatives is provided in or nearby the hospital	−4	−4	−4	−4	−4	−3	−4	−1**
33	Healthcare professionals involve relatives in decisions regarding the patient’s care	0	+1	−2**	0**	−1	−1	+1*	−2
34	Healthcare professionals pay attention to loved ones in their role as carer for the patient	−1	−1	−1	−1	0	0	−1	−1
35	Healthcare professionals pay attention to the needs of family and friends of the patient	−2*	−1*	−4*	−2**	−4	−2	−1	−3

*IC* Intensive care

**p <.01, *p <.05. Scores range between −4 and +4 (see Fig. 1)

### Viewpoint 1: “Treating patients with dignity and respect”

The general view expressed by respondents with viewpoint 1 was that provision of PCC required prioritization of patient preferences. Healthcare professionals with this viewpoint stated that “treating patients with dignity and respect” [statement (st.) 6, scored as +4 within this viewpoint] is a basic condition for healthcare provision and a foundation for every other aspect of care:

“Everybody should be treated with respect no matter what disease they have, what nationality, race or background, or whether they are a homeless person or a VIP, everybody should be treated with dignity and respect.” (Geriatrics Nurse 2)

“If there is no respect or if the patient feels that they are not being treated properly, they are not going to come back to you.” (Geriatrics Fellow 7)

The next most important statement also concerned patient preferences: “healthcare professionals involve patients in decisions regarding their care” (st. 9, +4). Healthcare professionals considered listening to the patient and incorporating their preferences and needs to be important principles of shared decision making. They argued that patients are the actual leaders of their care and should always be involved in the decision-making process:

“It is about engaging the patients, otherwise it is not PCC but completely physician directed. Making sure the patient is able to engage, and it is the healthcare professionals’ responsibility to make sure that the patient is involved.” (Quality Manager 1)

“Unless they are part of the decision, they won’t accept the treatment.” (Geriatrics Nurse 2)

“Patients are supported to set and achieve their own treatment goals” (st. 1, +3) and “healthcare professionals take into account patient preferences” (st. 16, +2) were also distinguishing statements for respondents with this viewpoint. These results further underscore the importance of patients’ preferences and involvement in decision making for PCC:

“Very often patients are not supported to set their own goals; it is the doctor telling them. Especially older patients … they are afraid to question authority and have an honest discussion about what they want and what they need. They are afraid to speak up. I don’t feel that a free and open dialogue is there as much as it should be.” (Quality Manager 1)

The information and education and coordination and communication dimensions were also ranked as important, as shown by the rankings for “patients are well-informed about all aspects of their care” (st. 30, +3), “patients know who is coordinating their care” (st. 27, +3) and “there is open communication between patient and healthcare providers” (st.14, +2):

“I think it will relieve some of their anxiety when they are better informed.” (Intensive Care Manager)

“One of the most important things, especially in the geriatric population with so many comorbidities, is that they know who is in charge so that they have a point person to go to when they are confused or if they have questions.” (Geriatrics Fellow 7)

In the information and education dimension, respondents felt that the main focus should be on communication and informing patients about all aspects of their care, rather than patients’ access to their care records (st. 24, −3).

Family and friends and access to care stood out clearly as the least important dimensions for this viewpoint on PCC. For instance, the relative unimportance of “accommodations for relatives are provided in or near the hospital” (st. 18, −4) was explained as follows:

“It would be great, but I do not think it is a priority. Making sure the patient is okay is our priority and if we find a place close for the family, great, but if not then not.” (Geriatrics Manager)

In addition, healthcare professionals from the geriatrics department argued that the provision of accommodations for relatives was not relevant for an outpatient practice. Respondents also ranked statements about access to care as less important, as shown by the ranking of “clear directions are provided to and inside the hospital” (st. 33, −4), “waiting times for appointments are acceptable” (st. 5, −3), “it is easy to schedule an appointment” (st. 10, −3) and “the hospital is accessible for all patients” (st. 7, −2):

“Most of the people will find their way.” (Geriatrics Physician 3)

“Patients do not mind waiting as long as everyone gets the care he or she needs eventually.” (Intensive Care Nurse 1)

In conclusion, the importance of the patient preferences, coordination, and information and education dimensions of PCC was distinctive for this viewpoint, which we labeled “PCC implies treating patients with dignity and respect.”

### Viewpoint 2: “Interdisciplinary approach”

Respondents with this viewpoint stated that healthcare provision using an interdisciplinary approach, in which coordination, patient preferences and information and education are the most important dimensions, is a central issue in PCC. “Healthcare professionals work as a team in care delivery to patients” (st. 3, +4) stood out clearly as the most important statement. Respondents indicated that healthcare professionals must always know what every other professional on a given team is doing at all times in order to provide care of the best quality. Furthermore, they argued that input from different specialties and professions is important and required for a comprehensive overview of a patient’s condition:

“You need all the information about a patient or you may not be aware of what the real problem is or recognize contributing factors to the patient’s condition.” (Quality Manager 3)

“I think patient care needs to be well coordinated between professionals; otherwise you lose the patient in the middle of the lack of coordination.” (Quality Manager 3)

In addition, information and education was considered an important dimension for PCC, evident form the high ranking of “healthcare professionals have good communication skills” (st. 15, +3) and “there is open communication between patient and healthcare professionals” (st. 14, +3):

“Bad communication, not ineffective and miscommunication is probably the most significant reason why errors happen.” (Quality Manager 3)

The patient preferences dimension was also found to be of importance, but in a more indirect, outcome-oriented manner than in viewpoint 1. The statement “healthcare professionals treat patients with dignity and respect” (st. 6, +3) was again ranked highly, but “healthcare is focused on improving the quality of life of patients” (st. 20, +4) was foremost:

“Sometimes we just want to treat the patient, but we are not sure whatever we are doing is going to improve the quality of life in a positive way. I think that this is most important.” (Intensive Care Fellow 4)

Family and friends appeared to be the least important PCC dimension. In accordance with viewpoint 1, “accommodations for relatives are provided in or near the hospital” (st. 18, −4) was ranked as least important for PCC. Additionally, “patients are in charge of their own care” (st. 29, −4) and “healthcare professionals support patients to be in charge of their care” (st. 13, −3) were considered to be among the least important statements:

“The patients are in charge of their care … but they should not be dictating basically what the care is, not knowing everything about the disease or other treatment options that are out there.” (Intensive Care Physician 3)

This perspective was particularly prevalent at the SICU:

“They should be involved, but I think patients here are usually more acute, they are sicker, so it is kind of out their hands at that point … When they are here, in the hospital, that might not be the time that they are dealing with all of that.” (Intensive Care Paramedic 2)

Although most respondents with this viewpoint argued that healthcare professionals should be well informed about a patient’s condition, they ranked “healthcare professionals are well informed; patients need to tell their story only once” (st. 12, −3) as one of the least important statements:

“I think that patients need to tell their story as many times as necessary and I think that different people in the continuum of their care need to hear that story.” (Quality Manager 3)

“When they tell their story to different people, different information comes out.” (Quality Manager 2)

In conclusion, this viewpoint emphasizes the importance of coordination and information exchange among professionals for high-quality care. We labeled this viewpoint “PCC implies an interdisciplinary approach.”

### Viewpoint 3: “Equal access and good outcomes”

Respondents representing the third viewpoint stated that equal treatment of all patients is essential for PCC provision. Respondents with this viewpoint emphasized the importance of access to care. “The hospital is accessible for all patients” (st. 7, +4) was ranked as one of the most important statements, which respondents explained by stating that everyone who needs care should be able to receive it, in terms not only of a patient’s physical ability to get to the hospital, but also of a general foundation in society:

“There was a time when a person did not have insurance, sometimes the hospital would turn them away. To me that is just morally incorrect. In a way it is like if you cannot pay, you can die.” (Geriatrics Nurse 5)

Furthermore, “language is not a barrier to access to care” [st. 22] was ranked significantly higher than in the other two viewpoints:

“No language should stop you from going to the doctor and getting the care you need. Because when you do not know what someone is telling you, how can you take care of yourself?” (Geriatrics Nurse 6)

The importance of this issue may reflect the great diversity of the population served by Mount Sinai Hospital, located between the Upper East Side and East Harlem.

The patient preferences dimension was also central in this third viewpoint on PCC. In addition to the statements “healthcare is focused on improving the quality of life of patients” (st. 20, +3) and “patients are well informed about all aspects of their care” (st. 30, +3), “healthcare professionals treat patients with dignity and respect” (st. 6, +4) was ranked highly by respondents with this viewpoint:

“An overall goal in medicine.” (Intensive Care Fellow 5)

“If we do not treat patients with dignity and respect, we are not really doing our job.” (Geriatrics Physician 4)

“Patients’ knowledge is important in their outcomes. If they feel they are in charge of their care and their body and … everything that is being done to them, it will be in their best interest and further their overall health.” (Geriatrics Physician 4)

This perspective resembles the outcome-oriented manner in which patient preferences played a role in viewpoint 2.

The needs of family and friends received low rankings: “accommodations for relatives are provided in or near the hospital” (st. 18, −4) was ranked as least important, followed by “healthcare professionals pay attention to the needs of the patient’s family and friends” (st. 34, −4). Respondents emphasized that although relatives are very important in a patient’s care process, the main focus of healthcare professionals should always be on the patient.

In conclusion, respondents representing this viewpoint emphasized that accessibility of care to any patient and a focus on patient outcomes are important for PCC. We labelled this viewpoint “PCC implies equal access and focus on patient preferences.”

### Subgroup analyses

Separate analysis of data from the two departments revealed two main viewpoints in the geriatrics department, represented by all 16 respondents, and three main viewpoints in the SICU, represented by 12 of 15 respondents. The corresponding idealized statement rankings are presented in Table 3. As indicated by the correlations (Table 4), the department viewpoints differed little from those of the overall sample. In the SICU, viewpoint 1 was correlated least with the other viewpoints, but interpretation of the factor provided no new perspective on the study topic. Nonetheless, several remarkable differences between departments regarding specific elements of PCC were evident. For instance, coordination of care appeared to be more important in the geriatrics department. In particular, many professionals in this department ranked “patients have a first point of contact who knows everything about their condition and treatment” (st. 17) as very important, whereas this statement seemed to be fairly unimportant in the SICU:

“I think this is very important for the geriatric population. They are so sick they may not be able to tell me all the details about what they have received or what is actually going on. Now healthcare is so fragmented that you only get bits and pieces. Having somebody that can call to get that information is really important in his or her care.” (Geriatrics Physician 1)

**Table 4:** Correlations between views

		View 1	View 2	View 3
View 1		1.00	0.73	0.54
View 2			1.00	0.59
View 3				1.00
Geriatrics Department	View 1	**0.90**	**0.81**	0.61
	View 2	0.42	0.59	**0.82**
Surgical IC Unit	View 1	0.56	0.66	0.71
	View 2	**0.90**	0.61	0.54
	View 3	0.62	**0.94**	0.65

*IC* Intensive care

Correlations >0.80 printed in bold

Professionals working at the SICU highlighted the importance of continuity of care, giving fairly high rankings to “when a patient is transferred to another ward, relevant patient information is transferred as well” (st. 35). The information and education and physical comfort dimensions seemed to be less important to SICU professionals, many of whom felt that “patients are in charge of their own care” (st. 29) and “healthcare professionals support patients to be in charge of their care” (st. 13) were least important. The lack of importance of these statements could be explained by SICU patients’ conditions; many are too sick to participate in the decision making process, as described by respondents holding viewpoint 2. Finally, SICU professionals ranked “patients in hospital have privacy” (st. 2) as less important. This could be significant for an ICU as well, as patients at the Mount Sinai Hospital SICU were treated in an open space and did not have private rooms.

These examples show that certain aspects of PCC may be of particular concern for specific departments, in addition to the more general viewpoints distinguished above.

## Discussion

This study explored the relative importance of eight dimensions of PCC from the perspectives of healthcare professionals working at the geriatrics department and SICU of Mount Sinai Hospital in New York City. Three main viewpoints on elements that are important for PCC were distinguished. Respondents representing the viewpoint that PCC implies “treating patients with dignity and respect” attached great importance to patient preferences, coordination, and information and education. Those representing the viewpoint that PCC implies “an interdisciplinary approach” emphasized the importance of coordination and information exchange among professionals for high-quality care. Finally, respondents representing the viewpoint that PCC implies “equal access and good outcomes” emphasized accessibility of care to any patient and a focus of care on patient outcomes.

Rathert and colleagues [2] argued that a constellation of interventions representing all eight dimensions of PCC is needed to improve the quality of care for patients. However, the results of this study suggest that healthcare professionals do not find all eight dimensions to be equally important for PCC. Moreover, we revealed different views regarding the importance of PCC dimensions, which seemed to be only partly related to the context of care provision. Overall, patient preferences appeared to be one of the most important dimensions for PCC, followed by information and education and coordination of care. The physical comfort, emotional support, and continuity and transition dimensions were found to be of intermediate importance for PCC, and the family and friends dimension was clearly the least important. The importance of the access to care dimension differed notably among PCC viewpoints. Most respondents agreed that the provision of directions within the hospital, scheduling of appointments and waiting time were not very important for PCC. Most also tended to agree that language should not be a barrier to access to care, which could be explained by the heterogeneous patient population that Mount Sinai Hospital serves. However, respondents had considerable differences of opinion regarding the statement “the hospital is accessible for all patients,” related to the interpretation of “accessibility” in physical or financial terms. Those who interpreted accessibility in physical terms – as intended (Table 1) – ranked it as of low importance for PCC, just as they ranked, for instance, the provision of directions within the hospital. Many professionals who felt that accessibility was less important assumed that patients would not have difficulty finding the hospital and that signage provided good directions inside and outside the hospital. This perspective could also be related to the location of Mount Sinai Hospital between the Upper East Side and East Harlem. One could reasonably assume that professionals working in hospitals where physical accessibility is less likely to be a matter of course would consider this statement to be more important for PCC. Those who interpreted it as financial accessibility to care, representing viewpoint 3, appeared to be concerned about equal access to and good outcomes of care for all. However, according to the definition used in this study, financial accessibility is not part of PCC; rather, it is part of “equitable care” [1]. This alternative interpretation of the accessibility statement was thus unintended and raises some issues. Methodologically, respondents’ attribution of different meanings to statements while expressing their views by ranking statement sets is a positive feature of the use of Q methodology to explore subjectivity. Generally formulated statements allow alternative interpretations that participants can use to express their perspectives; these interpretations may provide unexpected study data. On the other hand, general statements that allow for interpretations that are undesired or irrelevant in the context of a study may become a matter of concern. However, we feel that the inclusion of financial accessibility in the interpretations of participants expressing viewpoint 3 is not necessarily a matter of concern, as participants were asked to describe their views; if financial accessibility had explicitly not been part of the statement set, participants would still have bene free to express this concern during interviews. Theoretically, one could delimit concerns about the patient-centeredness of care to the group defined as patients, who by definition have access to care, excluding those who are in need of care but cannot access it for reasons such as race, ethnicity, gender, and income [1]. However, healthcare professionals in this study did not perceive PCC and equitable care as entirely separate. Moreover, financial accessibility to care may be a less pressing issue in contexts with different healthcare system funding e.g., [17]. Nevertheless, the unintended alternative interpretation of the accessibility statement in this study may complicate generalization of the results, particularly in the wider context of care quality, and as such should be interpreted as a weakness of this study. Future researchers wishing to explore perspectives on PCC using the statement set presented in Table 1 should thus consider carefully whether to revise the accessibility statement to focus specifically on physical accessibility (e.g., “The hospital is physically accessible for all patients”) or to use separate statements for physical and financial accessibility (e.g., “The hospital is physically accessible for all patients” and “Care is financially accessible for all patients”).

Subgroup analyses conducted to explore whether professionals’ viewpoints were related to the context of care provision showed no substantial difference between departments, but a number of interesting minor differences. For example, the importance of coordination of care for PCC clearly differed between departments. Respondents from the geriatrics department felt that a primary contact person who knows everything about a patient’s condition was fairly important for PCC, whereas those from the SICU felt that this element was of much less importance. This finding may be explained by the older, vulnerable patients served at the geriatric department; these patients have reduced physical, social and cognitive functioning, and require care for longer periods of time [24]. They would thus potentially benefit more from having case managers to coordinate their care. At the SICU, on the other hand, healthcare professionals’ teamwork in delivering care to patients, as well as continuity and transition, were considered of much greater importance for PCC. This finding may be explained by the transfer of nearly all patients attending the SICU to other wards before discharge, a situation that is much less common in the context of an outpatient practice, such as the geriatrics department. The lesser importance of patient privacy in the hospital among SICU professionals may be explained by the context; patients at the Mount Sinai Hospital SICU did not have private rooms. Finally, SICU respondents found the ability of patients to be in charge of their own care and to access their medical records to be less important for PCC, which may be rather typical for an ICU [25].

A remarkable finding of this study was that nearly all respondents, regardless of viewpoint or care context, ranked the treatment of patients with dignity and respect as most important for PCC. This finding could be explained by a general patient orientation among healthcare professionals, or perhaps (also) by the distinctive American custom of treating consumers with sympathy, dignity and respect, which is considered a civil rights issue [26]. Repetition of this study in other countries to explore whether this element carries equal importance elsewhere would thus be interesting. Another remarkable finding is that nearly all respondents explained that having patients tell their story more than once is important for PCC. To some extent, this finding contrasts with the results of some recent studies on the integration of care, which have shown that many patients consider repetition of their story a priority, although they find it to be tiring and frustrating [27].

This study has several limitations. First, it was conducted in a large American teaching hospital that serves a very diverse patient population. As type and context of care certainly appeared to affect the relative importance of the eight dimensions of PCC, replication of this study in other care settings and countries would be interesting. For example, Rathert et al. [2] showed that patient condition and self-management abilities affect the relationship between PCC and patient outcomes, which may indicate that patients in good and poor health, and/or those with good and poor self-management skills, have different needs. We thus recommend replication of this study among healthcare professionals in a hospital serving such different patient populations, as well as in settings with different degrees of commitment to PCC. Furthermore, macro-level differences may affect perspectives on PCC. Given the absence of universal healthcare coverage in the U.S., access to care may be more important to Americans than to populations in which all citizens are insured, such as the Netherlands. More often than citizens of other countries, Americans go without needed healthcare because of cost [28]. A study conducted recently in the Netherland using the same set of statements and methodology among patients and the healthcare professionals treating them in a hemodialysis department indeed showed that patients with end-stage renal disease agreed on the relative unimportance of access to care dimensions [18]. Second, perceptions of PCC may be influenced at the team level, as well as the department level. The two departments examined in this study comprised a single team. Future research is needed to investigate the effects of teams, departments, and organizational contexts on PCC viewpoints. Third, different choices could have been made in the design of this study. For instance, other researchers starting with the same eight dimensions of PCC may have developed a different set of statements; an obvious example would be choosing to phrase st. 18 explicitly in terms of physical accessibility of care. As discussed above, the alternative interpretation of this statement in terms of financial accessibility may have influenced our results, but apart from this specific point we have no reason to expect that an alternative set of statements representing the eight dimensions presented in Table 1 would lead to very different views on what is important for PCC in a similar care setting. Nonetheless, we would welcome replication of this study with an adjusted st. 18 to confirm these findings. Fourth, exploration of whether the joint findings of this study and future studies using the same research instrument could lead to the development of a scale to monitor PCC in healthcare organizations would be interesting. Such a scale should focus on the most important issues, as indicated by the views of relevant stakeholders. Whether such a scale should be care context specific or more generally applicable depends on the findings of future studies (such as those recommended above). Furthermore, use of such a scale would allow the investigation of the ideal way of providing PCC, beyond assessment of the current manner of care delivery. This approach could easily be achieved by simply changing the response categories. In Q methodology, respondents are asked about the most and least important aspects of the study topic based on subjective experience. Use of a survey would allow disentanglement of the prioritization of PCC aspects by distinguishing “ideal PCC” from “actual care delivery” resulting in so-called “gap” scores (calculated as the differences between “best care” and “current care”) [29, 30]. Finally, this study examined only professionals’ perspectives, and patients’ viewpoints may differ [31]. In a recently published study of PCC using the same methodology and statement set among patients with end-stage renal disease and professionals [18], however, two of four viewpoints were defined by professionals and patients, whereas the other two viewpoints were defined exclusively by patients. These findings support the notion that patients and professionals may have common and distinct views on PCC. The distinctions may results, for instance, from a difference in focus; care providers may define “care” more narrowly as “clinical treatment,” whereas patients may perceive care in a broader sense. However, given the specific focus of that study (i.e., care for patients on dialysis in a hospital in the Netherlands), these results are not easily transferable to other contexts. Thus, additional research involving professionals and patients in other settings is needed.

## Conclusion

This study showed that healthcare professionals working in the geriatrics department and SICU of a New York City hospital did not perceived all eight dimensions of PCC as equally important for the improvement of this type of care. Viewpoints on important elements for PCC appeared to differ more among professionals than between departments, but overall, the patient preferences, information and education, and coordination of care dimensions were considered to be most important for PCC. The method and results of this study could contribute to the improvement of PCC by helping organizations to target their quality management efforts toward dimensions of PCC that are considered important in their particular context of care. As patient satisfaction, resulting from PCC, has been shown to be associated with treatment compliance, such efforts may then contribute to better health outcomes, reduced readmissions and consultations, and, consequently, reduced healthcare costs [32–36]. Healthcare organizations wishing to improve PCC should thus consider examining the relative importance of PCC dimensions in their specific contexts of care provision, which may increase the efficiency of improvement. However, as our study sample is not representative and consisted only of professionals (not patients), the results cannot be generalized outside the sample. More research is needed to confirm our study findings. Furthermore, a recent systematic review seeking to define an integrative model of patient centeredness indicated that these dimensions are interrelated, rather than independent [37]. Although the relative importance of each PCC dimension may vary and the amount of investment made in each dimension may differ, minimum availability of all aspects is important to improve patient outcomes, as all dimensions are interrelated and dependent on each other.

## Availability of data and materials

Not applicable.
